# Photodynamic hyperthermal chemotherapy with indocyanine green in feline vaccine-associated sarcoma

**DOI:** 10.3892/ol.2015.3557

**Published:** 2015-08-03

**Authors:** MASAKI ONOYAMA, TAKESHI TSUKA, TOMOHIRO IMAGAWA, TOMOHIRO OSAKI, AKIHIKO SUGIYAMA, KAZUO AZUMA, NORIHIKO ITO, KAZUHIKO KAWASHIMA, YOSHIHARU OKAMOTO

**Affiliations:** 1 Onoyama Animal Hospital, Asago 669-5213, Japan; 2 Department of Clinical Medicine, School of Veterinary Medicine, Faculty of Agriculture, Tottori University, Tottori 680-8553, Japan; 3 Aino Animal Hospital, Fukuroi 437-0028, Japan

**Keywords:** feline vaccine-associated sarcoma, indocyanine green, photodynamic hyperthermal chemotherapy

## Abstract

The anticancer effects of photodynamic hyperthermal chemotherapy (PHCT), which consists of a combination of indocyanine green photodynamic hyperthermal therapy and local chemotherapy, have previously been reported. The present study investigated the effect of PHCT in six cases of feline vaccine-associated sarcoma (FVAS) following conservative surgical resection. No recurrence was observed in three out of six (50%) cases, while recurrence was observed in the remaining three cases. Of note, each feline with recurrences had previously undergone surgical resection more than three times, whereas those without recurrence had undergone no or one previous resection. In addition, the three animals in which there was no recurrence survived between 893 and 1,797 days following surgery. In conclusion, the results of the present study suggested that PHCT may be a candidate as a novel adjuvant cancer therapy for FVAS.

## Introduction

Although soft tissue sarcomas rarely metastasize, they are locally invasive and have a high rate of recurrence (1). Wide excision is the first therapeutic choice; however, recurrence is common, even following this approach. Therefore, the addition of therapies, including radiation, chemotherapy or a combinations thereof, were reported to have improved therapeutic benefits (2–5). Feline vaccine-associated sarcoma (FVAS) in particular exhibits aggressive local-infiltration tendencies and high recurrence rates. Therefore, treatment of this type of sarcoma with a combination of these major therapeutic modalities is advised (6–8).

Indocyanine green (ICG) generates heat when stimulated by light at a wavelength of 808 nm (9,10) and produces oxygen radicals when exposed to light at wavelengths of 600–800 nm (11). The use of ICG as a photosensitizer with a broadband light source, instead of a diode laser, in photodynamic therapy (PDT) has been established as a combination therapy consisting of PDT and hyperthermia therapy (HT), which was named photodynamic hyperthermal therapy (PHT) (12,13). Previous studies by the current authors have investigated the anticancer effects of PHT in experiments *in vitro* (12) and *in vivo* (13) and it has been demonstrated that the effects of local chemotherapy were enhanced by HT (14). Subsequently, a method was developed using local chemotherapy in combination with PHT, entitled photodynamic hyperthermal chemotherapy (PHCT), which has previously demonstrated positive results in the treatment of spontaneous soft tissue sarcomas in dogs and cats (15). The present study investigated the therapeutic effects of PHCT in six cases of FVAS.

## Materials and methods

### 

#### Animals

**Figure 1.**
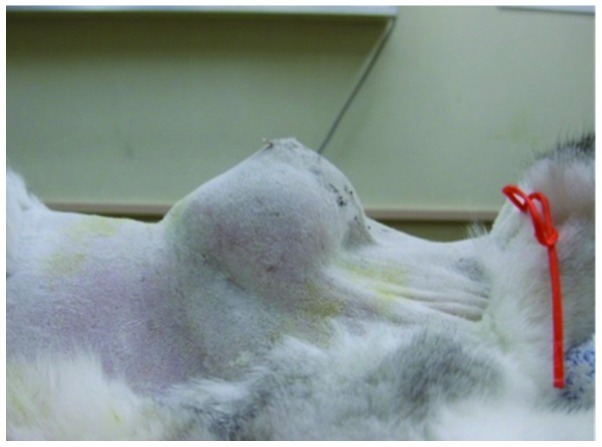
Tumor of a typical case of feline vaccine-associated sarcoma (case 1). Tumor is located at the interscapulum (4.5×4.0 cm; Right, cranial).

**Figure 2.**
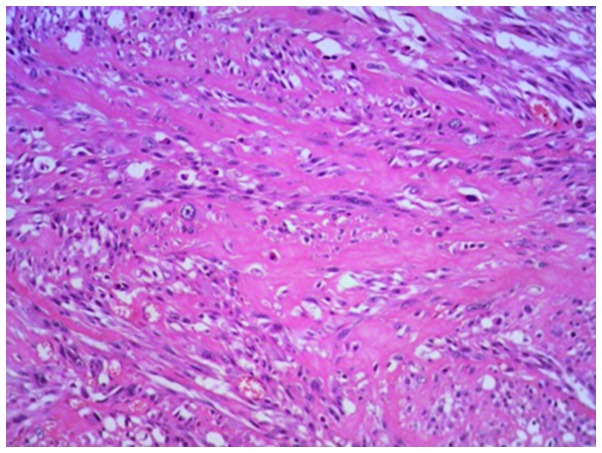
Representative histological image of feline vaccine-associated sarcoma (Case 1). Tumor tissues proliferate with collagen formation in certain areas and exhibit a fibrosarcoma-like histology. Hematoxylin and eosin staining; magnification, x200).

Table I presents a summary of the characteristics of the six cases of FVAS. The animals were presented for examination at the Aino Animal Hospital (Fukuroi, Japan) between August 2008 and May 2012. All six cats had a history of repeated vaccination in the dorsal region of the neck, interscapulum and scapular region. The ages of the animals ranged from 9 to 13 years. The breeds included five Domestic Short-Hair (DSH) cats and one American Short-Hair (ASH) cat. Tumors had developed in the dorsal thoracic region and had a maximum diameter range of 3–12 cm. No lung metastasis or bone resorption was observed on radiography scans in any case. Histopathological examination of preoperative biopsies or postoperative samples of the tumors yielded a diagnosis of soft tissue sarcoma (STS) or fibrosarcoma (FBS); in addition, all subjects were diagnosed with FVAS on the basis of clinical history and histopathological findings: Tumor tissues proliferate with collagen formation in certain areas and exhibit a fibrosarcoma-like histology (Figs. 1 and 2). In five out of the six cases, previous surgical resections had been performed between one and four times at a different veterinary hospital. Owners were informed about the risk of recurrence of this sarcoma, necessity of a wide and radical excision, probability of a functional illness, curative effect of combining surgery with other therapies, prognosis and financial burden. A combination of surgery, radiotherapy and chemotherapy performed at Azabu University Veterinary Teaching Hospital (Sagamihara, Japan) was proposed to the animal owners as the first therapeutic choice. However, the owners, desiring to minimize the side effects, invasiveness and stress of treatment, elected for treatment to take place at their regular clinic. Other treatments were then discussed with the owners, including the combination of PHCT and surgery. It was explained that PHCT was an experimental therapy and all owners of the pets enrolled in the present study provided written informed consent.

**Figure 3.**
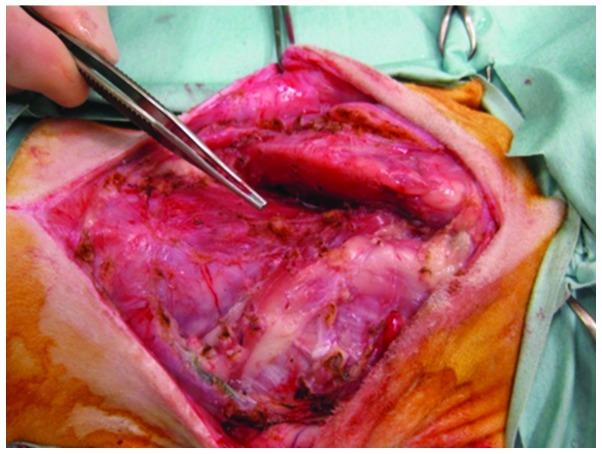
Representative image of the surgical field (Case 1) during tumor excision. The spinous process is not excised.

As the owners did not desire aggressive surgical resection, conservative resection with 2–3 cm surgical margins was performed. Excision involved regions of the neck and associated muscles, without partial scapulectomy or removal of the dorsal spinous processes; a representative image (Case 1), of the surgical field is shown in Fig. 3.

#### PHCT

**Figure 4.**
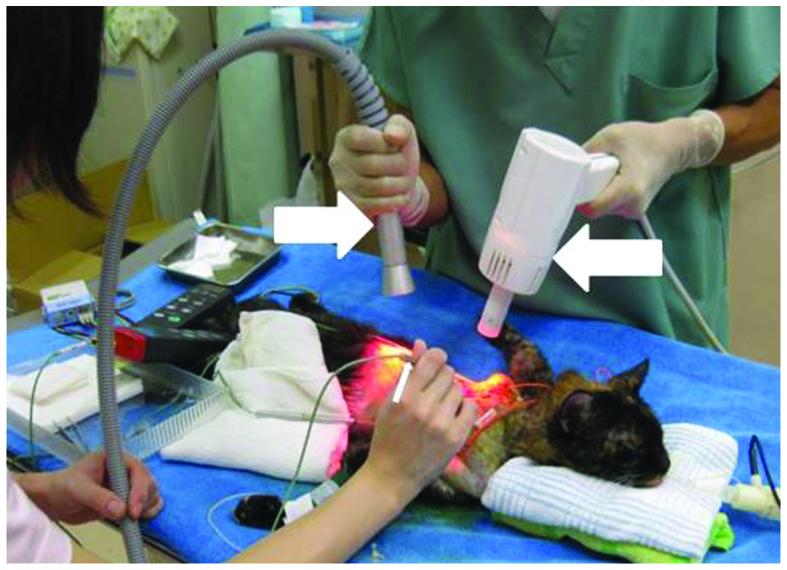
Typical image of the PHCT procedure (Case 2). PHCT was performed following surgery. Big arrows, the broadband lightsource; thin arrow, thermometer.

The PHCT procedure was performed as previously reported (15) under general anesthesia with isoflulene (DS Pharma Animal Health Co., Ltd., Osaka, Japan). In brief, ICG (25 mg/vial; Giagnogreen; Daiich Sankyo Co. Ltd, Tokyo, Japan) was dissolved in 9 ml saline (Otsuka Pharmacy, Co., Ltd., Tokyo, Japan) with an adjusted pH of 5.0. Anticancer drugs, including 1 ml carboplatin (50 mg/5 ml; Nippon Kayaku Co. Ltd, Tokyo, Japan) and 0.1 ml paclitaxel (300 mg/5 ml; Nippon Kayaku Co., Ltd). Tissue necrosis was observed in case 1 due to the paclitaxel treatment; as such, the volume of paclitaxel was reduced to 0.01–0.02 ml paclitaxel (30 mg/5 ml) in subsequent procedures. The solution was prewarmed at 45°C. A broadband light source (Super Lizer 5000; Tokyo Iken Co., Ltd, Tokyo, Japan), emitting a wavelength spectrum from 600 to 1,600 nm with a 5,000 mW maximum output power, was used. For each case, the tumors were resected and the ICG solution was injected into the resected area 3-dimensionally, including the skin surgical margin (2–3 cm), at a concentration of 1 ml per cm^3^ of the wound bed. Irradiation was applied at a distance of 10 cm from the resected area (irradiation area: 113 cm^2^, 40 mW/cm^2^) for 20 min per 113 cm^2^ (48 J/cm^2^) immediately following injection of the ICG solution, under general anesthesia with isoflulene. A representative image (Case 2) of the surgical procedure is shown in Fig. 4. The temperature at the surface of the resected area was monitored with a thermometer (Tokyo Iken Co., Ltd.) and was kept under 45°C by altering the proximity of the light source to the skin surface in order to maintain a uniform temperature at the radiation site; in addition, the interstitial temperature was maintained at 39.5–42.5°C.

The first round of PHCT was performed immediately following skin suturing post surgery. The treatment interval between the second and fourth round of PHCT was generally 1 week; treatment was then performed at intervals of 2–4 weeks. For the second and subsequent rounds of PHCT, the treatments were performed with all animals under either sedation or infiltration anesthesia using 3–5 ml/head of lidocaine (Xylocaine; AstraZeneca, Inc., Osaka, Japan). In all cases, follow-up examinations for recurrence and metastasis were performed at intervals of 2–3 months for one year following the first round of the PHCT. Thereafter, follow-up examinations were performed every 6 months.

## Results

The results of the present study are summarized in Table II. PHCT was performed between 6 and 20 times. The mean frequency of PHCT was 10.8 times (median, 10 times). The median disease-free survival (DFS) was 482 days (range, 30–1797 days). In three out of six (50%) cases (Cases 1, 4 and 5), no recurrences were observed for 893–1797 days following surgery; two of these cases had recurred following one previous surgery. Recurrence was observed between 30 and 70 days post surgery in the remaining three cases (Cases 2, 3 and 6); these cases had all undergone more than three surgical resections prior to PHCT. The three cats that exhibited cancer recurrence succumbed to progression of the tumor.

According to the outcomes of the subjects in the current study, the efficacy of the treatment was not suggested to be affected by treatment frequency. Although slight skin redness and minor skin burns occurred following PHCT, no severe side effects, including severe skin burns and necrosis, were observed in any of the animals except for Case 1. In Case 1, rupture of the skin sutures occurred due to an excessive volume of paclitaxel. The blood profile remained unchanged in all cases.

## Discussion

The present study revealed that combination therapy consisting of conservative surgery and PHCT in FVAS prevented recurrence of the tumor in the cases that had undergone no or one previous surgical resection. These results were comparable to those of a previous study (15).

Reported rates of local recurrence of FVAS following only conservative excision range from 35 to 59% (16–18). Radical surgical resection, including two muscle planes and 5 cm margins, resulted in clean margins in 97% of cases and a local recurrence rate of 14% (19). Recurrence rates of 26–52% have been reported for surgical excision combined with adjuvant therapies, including pre- or postoperative radiation and chemotherapy (6–8,16,20–24). A previous study reported that the median DFSs for animals with tumors treated with surgical resection by a general veterinarian and a veterinary surgical specialist were 66 and 274 days, respectively; in addition, the overall median reported DFS was 94 days (25). Furthermore, the median DFS for radical excisions, including hemipelvectomy, partial scapulectomy and removal of the dorsal spinous processes, was 325 days, whereas the median DFS with margins of <3 cm was 79 days (25). These previous studies indicated that it is difficult to prevent recurrence with routine surgical excision alone. By contrast, the therapeutic effect of chemotherapy alone is inadequate, as revealed by a previous study which reported a 39% rate of effectiveness and a median DFS of 84 days (range, 21–240 days) (20). Therefore, radiotherapy performed at the earliest possible time following surgical resection has been recommended for the treatment of FVAS (6–8,26), as median DFSs of 661 days (23–1109 days) (7) and 584 days (37–2490 days) (8) have been obtained with this treatment. Thus, this combined therapeutic approach is more effective compared with surgery alone. However, radiotherapy is not readily accessible to general practice veterinarians and owners due to the limited availability of radiotherapy facilities. Therefore, few animals may benefit from this treatment.

In the present study, the three cases (cases 2,3 and 6) that displayed cancer recurrence following PHCT had undergone three or more previous surgical resections and recurrence was observed earlier following PHCT in these cases. With regard to the histology of the tumor tissues resected in the present study, no difference in the characteristics of the malignancy was recognized among the cases with multiple recurrences (Cases 2, 3 and 6) and the cases with no recurrence with this treatment (Cases 1, 4 and 5). Of note, conservative surgeries were performed for all cases. The present protocol of PHCT was unable to prevent recurrence in all the present cases and cancer recurrences may have occurred due to a more extensive tumor cell invasion in the cats with recurrence compared with those without recurrence. Therefore, further investigation of certain factors, including anticancer drugs and the treatment interval, is necessary in order to prevent recurrence.

In conclusion, in the present study, the overall recurrence rate was 50% and the median DFS was 482 days; however, the recurrence rate was 0% in the incipient cases or those with only one previous recurrence. Compared with the results of previous reports, these results suggested that PHCT may have an equivalent effect to that of advanced treatments, including radiotherapy. In the present study, conservative excision was performed in order to preserve the basal region of the muscular tissue and spinous processes with 2–3 cm surgical margins. Therefore, it was suggested that the risk of recurrence may be equivalent or increased compared with previous studies. However, the median DFS of the animals treated with PHCT was greater than that previously reported for conservative surgical resection. As a result, PHCT may be considered a useful adjuvant therapeutic modality for the treatment of FVAS.

## Figures and Tables

**Table I.** Summary of six clinical cases of feline vaccine-associated sarcoma.

Case no.	Breed	Gender	Age	Size, cm	Histopathology	TNM
1	DSH	Male	10	4.5×4	STS	T3N0M0
2	DSH	Female	16	9×4	STS	T4N1bM0
3	DSH	Female	11	12×10	STS	T4N3M0
4	DSH	Male	9	6×6	FBS	T3N0M0
5	ASH	Female	12	4×4	STS	T3N0M0
6	ASH	Male	13	Multiple^a^	STS	T4N1M0

a >10 tumors of 0.5–3 cm in diameter were identified. TNM, tumor-node-metastasis stage; DSH, Domestic Short-Hair; ASH, American Short-Hair; STS, soft tissue sarcoma; FBS, fibrosarcoma.

**Table II.** Summary of treatment outcomes.

Case no.	Anti-cancer drug	No. of treatments	No. of previous surgeries	Recurrence	Disease-free survival (days)
1	PTX, CBDCA	6	1	No	893
2	PTX, CBDCA	11	3	Yes	34
3	PTX, CBDCA	10	3	Yes	70
4	PTX, CBDCA	10	1	No	1,526
5	PTX, CBDCA	8	0	No	1,797
6	PTX, CBDCA	20	4	Yes	30

PTX, paclitaxel; CBDCA, carbopulatin.
